# Extracellular Vesicle-Based Hybrid Systems for Advanced Drug Delivery

**DOI:** 10.3390/pharmaceutics14020267

**Published:** 2022-01-23

**Authors:** Diego A. Rodríguez, Pieter Vader

**Affiliations:** 1 CDL Research, University Medical Center Utrecht, 3584 CX Utrecht, The Netherlands; d.aguilarrodriguez@umcutrecht.nl; 2 Department of Experimental Cardiology, University Medical Center Utrecht, 3584 CX Utrecht, The Netherlands

**Keywords:** extracellular vesicles, synthetic nanoparticles, hybrids, drug delivery

## Abstract

The continuous technological advancement of nanomedicine has enabled the development of novel vehicles for the effective delivery of therapeutic substances. Synthetic drug delivery systems are nano-sized carriers made from various materials that can be designed to deliver therapeutic cargoes to cells or tissues. However, rapid clearance by the immune system and the poor targeting profile of synthetic drug delivery systems are examples of the pressing obstacles faced in nanomedicine, which have directed the field toward the development of alternative strategies. Extracellular vesicles (EVs) are nanoscale particles enclosed by a protein-rich lipid bilayer; they are released by cells and are considered to be important mediators of intercellular communication. Owing to their natural composition, EVs have been suggested to exhibit good biocompatibility and to possess homing properties to specific cell types. Combining EVs with synthetic nanoparticles by defined hybridization steps gives rise to a novel potential drug delivery tool, i.e., EV-based hybrid systems. These novel therapeutic vehicles exhibit potential advantageous features as compared to synthetic drug delivery systems such as enhanced cellular uptake and cargo delivery, immuno-evasive properties, capability of crossing biological barriers, and tissue targeting profile. Here, we provide an overview of the various strategies practiced to produce EV-based hybrid systems and elucidate those advantageous features obtained by synthetic drug delivery systems upon hybridization with EVs.

## 1. Introduction

Drug delivery science focuses on the development of novel vehicles for the effective delivery of therapeutic substances [1]. Synthetic nanoparticles (sNPs) are nanoscale particles of defined chemical composition and size which among their wide variety of applications (e.g., catalysis, imaging, bioremediation [2]) can also be designed as drug delivery systems (DDS). The molecular structure of the sNP surface as well as their particle size and shape determine properties such as sNP stability and solubility [3]. These properties can be customized by modifying the sNP chemistry, allowing the production of an extensive variety of formulations for drug delivery applications. Although sNPs can be derived from various materials, the most commonly used materials for drug delivery purposes are based on synthetic polymers or lipids [4,5,6,7,8]. The technological advancements in nanomedicine have allowed the development of improved synthetic DDS over recent years (e.g., through the addition of targeting ligands/proteins or by PEGylation); however, their poor stability, incapability of circumventing the immune system and crossing biological barriers, dose-limiting toxicity and immunogenicity, as well as a limited targeting profile remain limitations of concern present across the extensive variety of sNPs [9,10,11,12,13].

Extracellular vesicles (EVs) are nano-sized particles enclosed by a protein-rich lipid bilayer. They participate as important mediators of intercellular communication by carrying biological cargo including proteins, lipids, and nucleic acids between cells [14,15]. Interactions between EVs and recipient cells have been shown to modulate (patho)physiological processes in the body [16,17,18,19].

Tremendous effort has been placed on investigating their biogenesis, function, bio- and physicochemical composition and more recently, their possible therapeutic applications. EVs are typically classified based on their size and/or biogenesis: EVs of 30–150 nm in size produced upon the fusion of multivesicular bodies with the plasma membrane are termed exosomes; EVs of 50–1000 nm in diameter formed by budding of the cell membrane are referred to as ectosomes or microvesicles; and EVs encapsulating cellular material that are released upon apoptosis are known as apoptotic bodies, whose size varies from 50 nm up to a few micrometers [20]. Continuously, additional types of EVs are being discovered with overlapping properties, adding to the complexity of their classification [21]. In order to circumvent confusion caused by their heterogeneity, the International Society for Extracellular Vesicles (ISEV) suggests utilizing the general term “extracellular vesicle” for all particles that are naturally being secreted from cells, are enclosed by a lipid bilayer, and cannot replicate. More recently, operational terms for EV subtypes based on their size (e.g., small or medium/large EVs), biochemical composition, and cell of origin are encouraged to be implemented [21].

In recent years, EV-based DDS have gained substantial popularity due to their potential intrinsic benefits such as their suggested preferential accumulation to specific cells/organs, low immunogenicity [22,23], and suggested ability to cross biological barriers [24,25]—properties poorly found in synthetic DDS. Although it is not completely clear what attributes bestow EVs with such properties, it is speculated that the presence of specific molecules on the EV surface such as integrins, tetraspanins, and proteoglycans [26] may contribute to increasing the targeting specificity, enhancing cellular uptake, as well as avoiding recognition and rapid clearance by the immune system [27]. Thus, the naturally equipped protein-rich membrane of EVs is of utmost interest for the development of novel bioinspired nanoplatforms which could potentially outperform synthetic DDS. However, when developing EVs as DDS, some drawbacks can be listed such as the lack of standardized methods to efficiently load them with therapeutic cargo [28,29].

**Figure 1:**
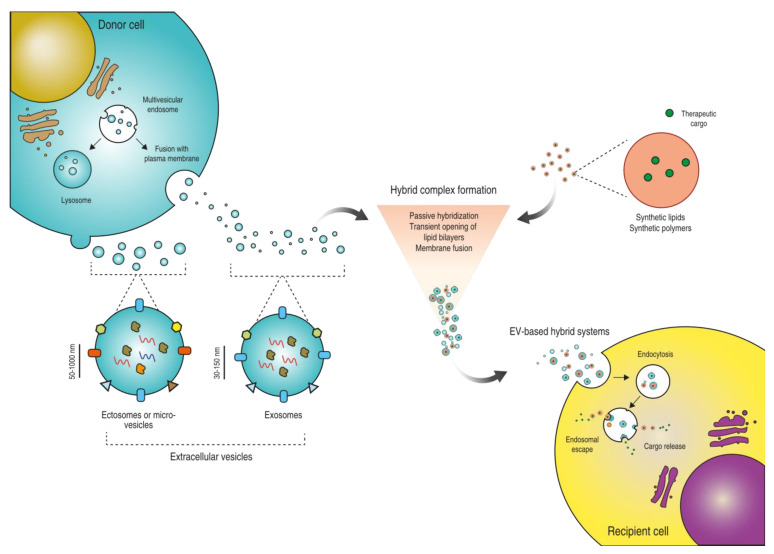
EV-based hybrid systems for drug delivery. Schematic representation of the biogenesis and release of EVs by the donor cell (light green). EVs produced upon the fusion of multivesicular endosomes with the plasma membrane or formed by budding of the cell membrane can be isolated and hybridized with synthetic nanoparticles (e.g., synthetic polymeric or lipidic nanoparticles loaded with a therapeutic cargo) through different production strategies in order to develop EV-based hybrid systems capable of delivering the cargo into recipient cells (yellow).

The combination of EVs with sNPs (e.g., polymers or liposomes conjugated/loaded with biological agents) by defined techniques results in the formation of a novel drug delivery nanoplatform, i.e., EV-based hybrid systems (Figure 1). Here, we elucidate the various strategies employed for the development of such EV-based hybrid systems, discuss their potential and demonstrated advantages in comparison to synthetic DDS and, lastly, conclude by sharing our opinions and future perspectives on the application of EV-based hybrid systems as advanced drug delivery vehicles.

## 2. Strategies to Prepare EV-Based Hybrid Systems

**Figure 2:**
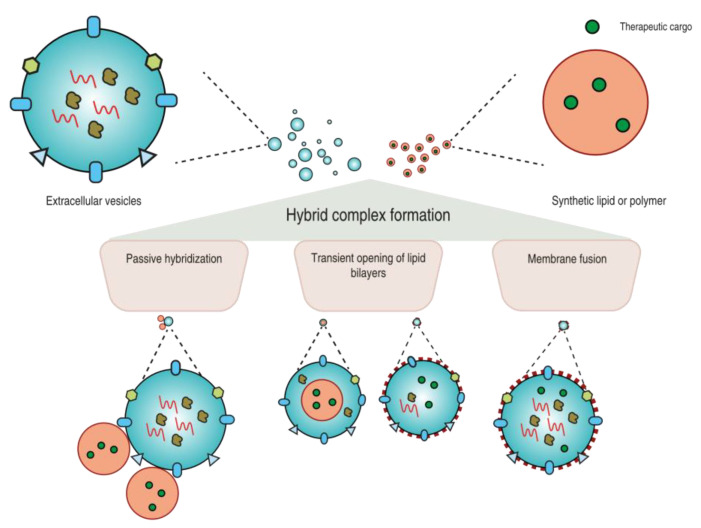
Production strategies for EV-based hybrid systems for drug delivery applications. Schematic illustration of the possible strategies to form EV-based hybrids with regard to final composition and architecture. Through passive hybridization, synthetic nanoparticles (sNPs) are most likely to interact with the EV surface or vice versa. The production strategy employing the transient opening of lipid bilayers (of either EVs or sNPs) may lead to multiple possible hybrid structures, which are determined by the sNP composition and hybrid formation conditions. Membrane fusion may allow hybrid formation without significant loss of sNP and EV cargo.

In recent years, techniques that were traditionally applied in other areas are currently being evaluated for the production of EV-based hybrid systems. Important considerations must be taken into account when selecting a production strategy, such as reproducibility, ability to control hybrid size and stability, possibility of eliminating possible by-products, and the preservation of the carriers’ functional characteristics. Generally, the techniques employed to prepare EV-based hybrid systems are based on physicochemical methodologies whereby the two nanoparticles (EVs and sNPs) are hybridized by means of electrostatic/hydrophobic interactions through the transient disruption/permeabilization of the lipid membrane which upon reassembly results in a hybrid complex formation or through fusion between lipid layers (see Figure 2). The combination of two distinct production strategies has also been implemented in an attempt to hybridize EVs and sNPs more efficiently. In this section, we will recapitulate the various strategies that are currently utilized to form EV-based hybrid systems.

### 2.1. Passive Hybridization

The principle to form EV-based hybrid systems by passive hybridization is relatively simple in comparison to other strategies practiced. Perhaps its greatest advantage is the presumable retention of the EV membrane integrity upon hybridization, as no “harsh” physical methods are applied. The physicochemical composition of both DDS (EVs and sNPs) is exploited to generate EV-based hybrids via electrostatic or hydrophobic interactions. As for the former, the hybrid formation is crucially dependent on the surface charge of both EVs and sNPs. In this regard, as the EV membrane is negatively charged, it can interact with cationic sNPs upon mixing, resulting in the formation of a newly hybridized nanoparticle. In a recent example, EVs obtained from bovine colostrum powder were complexed with polyethyleneimine (PEI) through electrostatic interactions, after which nucleic acids (either siRNA or pDNA) were added [30]. The purified EV-based hybrid systems exhibited unaltered properties in terms of size and polydispersity index as compared to unmodified EVs. The principle of forming hybrids by passive hybridization was also demonstrated by mixing lipoplexes (i.e., lipofectamine complexed with pDNA) with EVs, which resulted in the successful association of even large plasmids such as the CRISPR/Cas9 expression vector [31].

Alternatively, the EV membrane can be utilized as an anchor point where sNPs can interact with via hydrophobic interactions. For example, magnetic nanoparticles and cationic nanogels have been successfully conjugated to EVs through the insertion of their cholesteryl groups into the lipid bilayer of EVs, resulting in ≈80% of the EVs and magnetic nanoparticles being complexed and forming stable nanoparticles within 24 h of incubation [32,33].

Although these examples demonstrate that the generation of EV-based hybrid systems is feasible by exploiting the physicochemical properties of EVs, a potential disadvantage of this strategy may be that its use is limited to sNPs with specific surface charge or composition, the possible presence of unwanted by-products, as well as the lack of having a controlled size of the resulting EV-based hybrids.

### 2.2. Transient Opening of Lipid Bilayers

The production strategy for generating EV-based hybrid systems via sonication, freeze–thawing, or extrusion shares a common principle: transient disruption of the lipid layers of EVs and in some instances also of sNPs that then reassemble, resulting in the formation of hybrid systems (see Figure 2). In sonication, high-frequency sound waves are used to temporarily disrupt the structure of the lipid layer, whereas the freeze–thaw method gives similar results through the formation of ice crystals. Extrusion also disrupts EV/sNP lipid bilayers transiently, but in this case, it is due to the physical forces applied when the samples are extruded through a membrane of defined pore size. 

For instance, polyplexes of siRNA complexed with PEI have been hybridized with EVs isolated from various cancer cells [34]. In contrast to liposome–PEI complex formation [35], it was argued that the simple mixing of EVs and PEI would be insufficient to form stable and functional EV-based hybrid systems; thus, the assistance of sonication was subsequently implemented during assembly and compared to unsonicated hybrids. In this particular example, the combination of two production strategies was incorporated: electrostatic interactions and the transient opening of lipid bilayers. Interestingly, the sonicated EV-based hybrids was the only group that retained knockdown efficacy even after 5 days of storage at room temperature as compared to polyplexes and unsonicated hybrids, suggesting that upon sonication, the functionality and/or stability of the hybrid system was increased [34]. As suggested by the authors, it is probable that the membrane of EVs serves as a “shell” that surrounds the polyplex, resulting in a more stable and functional EV-based hybrid system.

The ability to artificially manipulate fluids under controlled conditions at micrometric scales is known as microfluidics. Due to the possibility of producing DDS under more controlled conditions such as flow rate, mixing ratio, and temperature [36], microfluidics has gained substantial popularity in the field of nanomedicine [37,38]. Although this technology has been previously applied to generate cell membrane-coated nanoparticles [39,40], the production of hybrids of EVs and sNPs via microfluidic mixing was not introduced until recently [41,42]. Here, the assembly strategy was based on the combination of microfluidic mixing and sonication using a bath sonicator, as it was hypothesized that hydrodynamic forces upon mixing were not sufficient to transiently disrupt the membrane structure of EVs [41,42]. In fact, the importance of sonication was tested on hybrids made of EVs and poly(lactic-co-glycolic acid) (PLGA) with and without sonication, which showed that ≈90% of the sonicated EV–PLGA hybrids architecture exhibited a typical “core–shell” structure (PLGA nanoparticles surrounded by an EV membrane) in comparison to only ≈47% for the unsonicated samples. In addition, the sonicated EV–PLGA hybrids exhibited a smaller size, lower polydispersity index, and greater colloidal stability in comparison to unsonicated samples, which gradually increased in size up to ≈450 nm after 5 days of storage. Perhaps the fact of having a one-step production strategy with controlled conditions, while ensuring reproducibility, are the foremost advantages when utilizing microfluidics to produce EV-based hybrid systems for drug delivery applications.

Freeze–thawing is typically employed in nanomedicine to load liposomes with water-soluble molecules by transiently disrupting the lipid layer upon the formation of ice crystals [43]. Inspired by this, this strategy has also been tested to produce EV-based hybrid systems in recent years [44,45,46]. To our knowledge, the first approach of producing an EV-based hybrid system via freeze–thawing was performed by fusing EVs with various liposomal formulations [44]. The hybridization efficiency was seemingly dependent on the number of freeze–thawing cycles employed regardless of the lipid formulation tested, as all of the formulations showed relatively high fusion efficiencies. However, a heterogeneous size distribution was observed for all EV-based hybrids. Later, more researchers followed a similar production strategy, adding key and novel elements to their advanced EV-based hybrid systems [45,46]. For instance, EVs isolated from genetically modified cells overexpressing CD47 hybridized with thermosensitive liposomes loaded with a photothermal agent and an immune adjuvant have been used as a combined photothermal therapy and cancer immunotherapy [46]. These EV-based hybrids exhibited great colloidal stability even in the presence of fetal bovine serum for 7 days, as no changes in size were observed over time. Despite these encouraging examples, the exposure time and frequency, as well as the number of freeze–thaw cycles needed to efficiently open up the lipid membrane of EVs/sNPs, which is essential to allow subsequent hybridization or loading of a therapeutic cargo, while maintaining their biological integrity, is still largely unknown and requires further investigation.

Extrusion seems to be the preferred method to produce EV-based hybrids with synthetic lipids [47,48,49,50,51]. Given the membranous composition of EVs, lipids present in sNPs, such as lipid nanoparticles or liposomes, can be merged into the EV lipid bilayer by extruding EVs and sNPs together through membranes with defined pore sizes. As an example, it has been recently shown that EVs and siRNA can be incorporated at the hydration step of the lipid film hydration method for the preparation of liposomes, which is followed by subsequent extrusion steps to form stable EV–liposome hybrids loaded with siRNA [49]. Interestingly, although the encapsulation efficiency of siRNA into hybrids was slightly decreased as compared to liposomes, it was still substantially higher than what has been demonstrated for other EV-loading strategies [49]. The combination of sonication and extrusion has also been employed to produce EV-based hybrid systems [47,51]. In these two examples, EVs and sNPs were first mixed at defined ratios followed by a sonication treatment, after which several extrusion steps were performed. The EV-based hybrids exhibited a spherical morphology without any alterations in the architecture of EVs reported, suggesting that the sonication–extrusion treatment could also be a feasible production strategy for the development of EV-based hybrid systems. Perhaps, this combined production strategy might result in a more adequate mixing of both EVs and sNPs as compared to simple mixing prior to extrusion, but this requires further examination. 

Overall, the possibility of controlling hybrid size is what makes extrusion potentially advantageous in comparison to other methods. However, its limitation is that extrusion leads to a considerable loss of material, thus requiring high amounts of EVs if intended to be applied in a clinical setting. Importantly, transiently opening the EV architecture in an attempt to hybridize the lipid bilayer of EVs with sNPs is naturally more disrupting than passive hybridization and might require the careful manipulation and subsequent characterization of EV-based hybrids in order to ensure that the integrity and functionality of EVs is maintained [52].

### 2.3. Fusion of Lipid Bilayers

Producing EV-based hybrids by applying harsh methodologies, although not yet fully proven, might result in alterations and/or a possible loss of biological cargo of EVs. To avoid this, an interesting production strategy involving the membrane fusion of EVs and liposomes without leakage or disruption has been proposed [53]. This method relies on the PEG-induced fusion of lipid bilayers. As a strategy to avoid variations in regard to the lipid biocomposition of EVs upon fusion, the main lipids found in natural membranes were selected to produce liposomes. Interestingly, the fusion efficiency was evidently dependent on the PEG concentration, increasing from a lower to higher degree of hybridization with increasing PEG concentration. Although at slightly lower fusion efficiency, this PEG-dependent concentration behavior was also observed for EVs isolated from another cell type. In addition, the full membrane fusion of EVs and liposomes was suggested to occur, which means that both outer and inner lipid layers were fused, perhaps giving rise to the most advantageous property of this production method, which is the possibility of forming hybrids without detectable leakage of EV cargo.

Collectively, we have learned that the employment of strategic physicochemical techniques allows for the production of EV-based hybrid systems with reported improvements such as increased colloidal stability in comparison to synthetic DDS. Perhaps more detailed characterization studies of the EV-based hybrid systems could bring some clarity with regard to the final structure and composition of the newly formed nanoparticles. Lastly, developing production strategies with more controlled or automated settings might support reproducibility and avoid batch-to-batch variations of EV-based hybrid systems, which can be considered critical parameters for clinical therapeutic applications.

## 3. Advantages of EV-Based Hybrids in Comparison to Synthetic Drug Delivery Systems

In this section, we provide insight into the beneficial effects attributed to the addition of EVs to sNPs in vitro and in vivo, hypothesize the possible mechanisms by which hybrids might improve or outperform synthetic DDS, and discuss their potential for advanced drug delivery applications (see Table 1).

### 3.1. Immuno-Evasive Benefits

Recognition and rapid clearance by the immune system, as well as a short circulation half-life, might be the most challenging obstacles that sNPs encounter [54]. Therefore, novel approaches to reduce sNP elimination by the immune system are needed. Unlike sNPs, EVs may express surface proteins such as CD47, CD55, and CD59, which can potentially contribute to circumvent these limitations [55]. For instance, CD47 is known to orchestrate a protective mechanism by which both (cancer) cells and EVs evade phagocytosis as it interacts with its receptor signal regulatory protein alpha (SIRPα) present on immune cells to trigger a “do not eat me” signal [56]. In addition, CD55 and CD59 may protect EVs from complement-mediated degradation and increase stability in circulation [57]. Given this naturally present protective mechanism, EV surface characteristics could in principle confer immune-evasive properties and increase circulation time on sNPs upon hybridization [46]. For example, when cancer cell-derived EVs were hybridized with PLGA nanoparticles by the assistance of microfluidic sonication, macrophage-mediated uptake of the resulting EV-based hybrids as well as the immune response in mice was significantly lower as compared to that of cancer cell membrane- or synthetic lipid-coated PLGA nanoparticles [41], while the blood circulation half-life of such hybrids was shown to be ≈3.5-fold longer as compared to synthetic lipid-coated PLGA nanoparticles [42]. Similarly, gold nanoparticles first coated with branched PEI (AuBPEI sNP) and then coated with EVs isolated from cancer cells also showed significantly lower uptake by macrophages in comparison to AuBPEI sNPs without coating [48]. This evidence indicates that sNPs can inherit the immune privileged properties from EVs upon hybridization, which are possibly mediated by the demonstrated capability of EV-based hybrid systems to competitively interact with SIRPα present on macrophages [46]. Nevertheless, it is noteworthy that the EV-based hybrid systems discussed above were developed using EVs from various cancer cell lines that are known to bear overexpressed levels of CD47 on their surface membrane [58], which could explain how EVs equipped sNPs with immune protection properties. For this reason, careful attention is recommended with regard to the selection of EV source, as the immune-protective properties may differ between EVs isolated from different cell types. Additionally, isolating EVs from genetically modified cells overexpressing specific bioactive molecules including surface proteins, receptors, or ligands could contribute to enhancing the immune-protective mechanism of EV-based hybrid systems, allowing us to develop more precise and safe therapies. Collectively, EV-based hybrid systems represent an attractive alternative to synthetic DDS as they are decorated with EV-inherited bioactive molecules and therefore may be capable of escaping from immune surveillance and prolonging circulation half-life.

### 3.2. Overcoming Biological Barriers 

As a natural mechanism of protection against pathogens and diseases, biological barriers exert their role to safeguard the integrity of the body. Numerous biological barriers can be found in the human body that provide protection to specific tissue regions and organs [59]. However, this also makes the delivery of drugs to these cells/tissues more complicated. Varying results have been observed over the past years when utilizing sNPs as DDS aiming to penetrate biological barriers in order to exploit their therapeutic effects [60]. However, the therapeutic access of sNPs has been mainly hindered due to an inability to reach target cells in vivo [59]. In addition, sNPs may be degraded upon their entrapment in acidic compartments of the endo-lysosomal pathway [61], resulting in another impediment of their application. Conversely, evidence suggests that EVs possess the capability to cross biological barriers such as the blood–brain barrier [62] and release their cargo via membrane fusion with endosomes/lysosomes [63]. Since EVs may exhibit such great capability of crossing biological barriers, many researchers are inevitably allured by their promising potential as DDS [64].

The blood–brain barrier does not only impede the penetration of small molecules but also of therapeutic nanoparticles [65,66,67]. Thus, the presence of specific surface proteins on EVs may be more important to cross the blood–brain barrier than only their nanoscale size [62]. For instance, EVs derived from brain endothelial cells exhibited an increased transport across the blood–brain barrier in zebrafish as compared to EVs derived from other brain tumor cells, which is possibly mediated by the enriched presence of the surface protein CD63 [68]. Other types of tetraspanins have also been associated with enhanced cell penetration and/or fusion [69], whereas other surface proteins may assist EVs to access densely condensed tissue regions such as fibrotic stroma [70]. Inspired by this, novel EV–liposome hybrid systems were designed and produced through the fusion of EVs derived from fibroblasts with liposomes containing clodronate (EV-L-CLD) or not (EV-L). Both hybrids exhibited greater capability to penetrate pulmonary fibrotic tissue in mice (3.4- and 1.8-fold, respectively) as compared to liposomes [51]. Since the biodistribution of EVs in vivo seems to be affected by the cell source [71], it can be speculated that the enhanced interstitial penetration of EV-L hybrids may be due to the presence of specific proteins on the EV membrane. Thus, exploiting the intrinsic capability to cross biological barriers of EVs may assist in the precise delivery of therapeutics to biologically protected regions upon hybridization.

### 3.3. Enhanced Cellular Uptake and Cargo Delivery

Traditional synthetic nanoparticle formulations can be modified through e.g., PEGylation in an attempt to avoid unspecific cellular uptake and increase circulation half-life [72]. However, the PEGylation of NPs also results in lower cellular uptake and delivery efficiency. Therefore, alternative strategies are needed for the effective internalization of nanoparticles and subsequent cargo delivery [73]. Although the mechanism by which EVs are internalized efficiently by certain cell types remains unclear, there is literature which supports that it might be due to the presence of surface proteins such as adhesion molecules, transmembrane, and/or transport/fusion proteins [74], which may facilitate the adhesion of EVs to the cell membrane and/or promote a rapid initiation of cellular uptake [75]. In fact, increasing evidence suggests that EV may deliver their cargo more efficiently than synthetic DDS [73,76,77]. With this in mind, in an attempt to increase the internalization and cargo delivery of synthetic DDS, sNPs may be hybridized with EVs, speculating that the EV-based hybrid systems would inherit the aforementioned properties found on EVs. Indeed, multiple studies have shown a consistent tendency toward a significant enhanced internalization of EV-based hybrids as compared to their synthetic counterparts [41,45,46,51]. In one example, the possible mechanism by which EV-PLGA hybrids are internalized by cells was found to be primarily via clathrin-mediated endocytosis, which differed from PLGA NPs coated with cell membranes or synthetic lipids [41]. In addition, the acute depletion of cholesterol, which is crucial for lipid raft formation in cell membranes, led to an impaired uptake of SKOV3-derived EVs hybridized with polyplexes (siRNA/PEI) but not of the polyplexes alone, suggesting that the addition of EV surface features can alter sNP internalization mechanisms [34]. Novel strategies to investigate the internalization mechanism of EV-based hybrids are awaited with great interest and could aid in developing customized hybrids by, for example, overexpressing specific proteins to obtain a higher cellular uptake and/or even more functional delivery of therapeutic cargoes.

Mesenchymal stem cells (MSCs) are relatively resistant to transfection by sNPs, including lipofectamine [31]. To overcome this, an EV-based hybrid system was recently presented by which even a large plasmid could not only be successfully complexed but also delivered more efficiently than by lipofectamine alone [31]. Interestingly, pre-treating the EV–liposome hybrids with proteinase K resulted in a significant impairment to deliver the cargo in MSCs, indicating that retained EV membrane proteins upon hybridization are crucial for efficient cellular uptake and cargo delivery.

Increased nucleic acid delivery efficiency has also been demonstrated by others. EV-based hybrids made from bovine colostrum powder EVs and polyplexes (siRNA/PEI) are taken up more efficiently by lung and pancreatic cancer cells as compared to polyplexes alone [30]. In addition, lung cancer cells treated with these hybrids loaded with siRNA targeting KRAS exhibited a four-fold higher KRAS knockdown as compared to cells treated with polyplexes. The versatility of this novel EV-based hybrid system was further demonstrated by transfecting lung cancer cells with hybrids loaded with pDNA.

EVs have been suggested to preferentially interact with cells of their origin [77]. However, this was not observed in a cross-over experiment comparing hybrids made of EVs isolated from various cell types in different cell lines. Here, EVs from different cell types were isolated to produce hybrids with polyplexes (siRNA/PEI) with the assistance of sonication [34]. Although in this example, the cellular uptake was not particularly investigated but rather the silencing efficacy of the various hybrids, the results obtained were considerably variable without suggesting any preferential interaction to recipient cells of EV origin. Further investigation might bring some clarity as to whether these particular EV-based hybrids are preferentially taken up by certain cell types as compared to polyplexes or simply the knockdown efficiency of hybrids is more determined by intracellular trafficking differences among cell types.

### 3.4. Homing Properties

Unmodified sNPs have shown considerably lower targeting efficacy as DDS in comparison to those of natural origin such as EVs [78,79]. Although the underlying mechanism by which EVs display homing properties remains elusive, there is evidence that suggests that EVs may possess a preferential homing by ligand–receptor interactions [80], in particular by their integrin profile, which might also direct the biodistribution of EVs in a tissue-specific manner [81]. Perhaps, inheriting the innate homing capability of EVs [62,80,82] could be a most advantageous factor that sNPs may obtain upon hybridization with EVs, subsequently contributing to an enhanced delivery of therapeutics at the site of interest. As an example, in the study discussed above, EV-L accumulated to a larger degree in lungs than unmodified liposomes, which was attributed to the homing affinity of EVs to the pulmonary fibrotic tissue [51].

Stromal cell-derived factor 1 is abundantly present in the bone marrow niche and can interact with the CXCR4 receptor, promoting the recruitment of peripheral CXCR4^+^ hematopoietic stem cells to the bone marrow [83,84]. On that account, the capability of EV-based hybrids to target bone marrow was investigated using EVs isolated from cells overexpressing CXCR4 and fused with liposomes [50]. Different ratios of EVs to liposomes were injected in mice via the tail vein, showing that animals treated with EV-based hybrids exhibited a stronger fluorescent signal in the femora as compared to those treated with liposomes, which almost exclusively accumulated in liver. Interestingly, increasing the EV to liposome ratio also influenced the homing capability of EV-based hybrids, whereby those containing a higher ratio of EVs exhibited a greater targeted profile to bone marrow than those with lower ratios, which was possibly mediated by the inherited homing properties of EVs bearing overexpressed levels of CXCR4.

EV-mediated homing to tumors has also been reported [42]. Here, in an MDA-MB-231 tumor model in mice, hybrids were generated using MDA-MB-231-derived EVs and PLGA nanoparticles conjugated with the nucleolin-targeting aptamer AS1411 (AP EV–PLGA) or random-sequence oligonucleotides (Rdm EV–PLGA), or without conjugation (EV–PLGA), and compared to aptamer-modified synthetic lipid-coated PLGA nanoparticles (AP lipid–PLGA). Ex vivo evaluation of tissue distribution showed that the accumulation of AP EV–PLGA hybrids in the tumor was 12.5-fold higher as compared to AP lipid–PLGA but also 1.59-fold and 1.52-fold higher in comparison to EV–PLGA and Rdm EV–PLGA, respectively. The authors attributed the remarkable homing capability of the AP EV–PLGA hybrids to the incorporation of the aptamer (AS1411), which allowed specific binding to nucleolin present on tumor cell membranes as well as to the homologous origin of EVs and target tumor cells.

The question as to whether the homing capability of EV-based hybrid systems is mediated by the presence of specific surface proteins (either naturally present or artificially incorporated), or simply because of an increased circulation half-life and thus an increase in chances to accumulate at the site of interest, remains open for discussion. Nonetheless, cumulative evidence has demonstrated that EV-based hybrid systems hold great potential for the targeting of specific cells and tissues.

## 4. Concluding Remarks and Future Perspectives 

Producing EV-based hybrid systems represents a promising alternative to synthetic DDS due to the reported improvements obtained upon hybridization with EVs, including greater colloidal stability, enhanced cargo delivery and targeting profiles, and immuno-evasive properties. The development of EV-based hybrid systems has been tackled by different angles, seeking for the most suitable formulation capable of delivering therapeutic agents in a safe and efficient manner. The ideal source of EVs should have low immunogenicity, great scalability, and yield EVs capable of delivering therapeutic cargo to the target cell. However, the response of recipient cells to EV-based hybrid systems from different sources may also vary, which is why the most optimal EV source should be experimentally validated. Undoubtedly, the steps ahead are still challenging, as selecting an adequate source of EVs together with suited production strategies while guaranteeing safe treatments and ensuring batch-to-batch reproducibility may be the main points that require further investigation and improvement. Altogether, EV-based hybrid systems could allow the generation of more specialized drug delivery therapies in the future.

## Figures and Tables

**Table 1:** Overview of various strategies employed to produce EV-based hybrid systems as well as the attributed benefits.

EV Source	Isolation Method	Nanoparticle	Hybrid Formation Strategy	Therapeutic Cargo	Benefits upon Hybridization with EVs	Reference
L-929	Ultracentrifugation	Liposomes	Sonication and extrusion	Nintedanib	Enhanced cellular uptake Reduced accumulation in liver and enhanced penetration inside pulmonary fibrotic tissue	[51]
3T3 and A549	Ultracentrifugation	Liposomes	Sonication and extrusion	siRNA loading via electroporation	-	[47]
Bovine colostrum powder	Ultracentrifugation	Folic acid-coated EV + polyethyleneimine	Passive hybridization	siRNA and pDNA	Enhanced cellular uptake, gene silencing ability, and pDNA delivery in vitro	[30]
NIH-3T3 (overexpressing CXCR4)	Ultracentrifugation	Liposomes	Extrusion	antagomiR	Selective accumulation in bone marrow Increased miRNA silencing in vitro and in vivo	[50]
4T1	Density gradient, size exclusion chromatography	Gold nanoparticles	Extrusion	-	Reduced uptake by macrophages	[48]
PC3, SKOV3, HCT-116, Saos-2	Ultracentrifugation	Polyethyleneimine	Sonication	siRNA, anti-miRNA	Increased gene delivery efficacy and storage stability in vitro	[34]
CT26 (overexpressing CD47)	Ultracentrifugation	Thermosensitive-liposome	Freeze–thaw	ICG and R837	Enhanced cellular uptake and targeting capability Prolonged circulation time	[46]
BALB/c 3T3 (overexpressing CD47)	Ultracentrifugation	Thermosensitive-liposome	Freeze–thaw	Granulocyte-macrophage colony-stimulating factor, and/or docetaxel	Preferential accumulation in tumor and inhibition of tumor progression Enhanced cellular uptake	[45]
A549	Ultracentrifugation	PLGA	Microfluidics + sonication	-	Reduced uptake by macrophages Enhanced cellular uptake Homotypic targeting in vivo	[41]
MDA-MB-231	Ultracentrifugation	PLGA/Cholesterol-AS1411 aptamer	Microfluidics + sonication	-	Reduced uptake by macrophages Prolonged circulation time Increased accumulation in tumor sites	[42]
HUVEC, murine MSC	Ultracentrifugation	Liposomes	Membrane fusion	mTHPC	-	[53]
Raw264.7, CMS7-wt, CMS7-HE (overexpressing HER2 receptor)	Differential centrifugation and microfiltration	Liposomes	Freeze–thaw	-	-	[44]
SKOV3, CPC	Tangential flow filtration, size exclusion chromatography	Liposomes	Extrusion	siRNA	Reduced toxicity Intrinsic regenerative properties	[49]
HEK293FT	PEG 6000 precipitation method	Liposomes	Passive hybridization	pDNA	Functional delivery of large plasmids into MSCs	[31]

## Abbreviations

Extracellular vesicles	EVs
Synthetic nanoparticles	sNPs
Drug delivery system	DDS
International Society for Extracellular Vesicles	ISEV
Polyethyleneimine	PEI
Poly(lactic-co-glycolic acid)	PLGA
Poly(ethylene glycol)	PEG
Signal regulatory protein alpha	SIRPα
Mesenchymal stem cells	MSCs
